# Morphology Changes in Human Fungal Pathogens upon Interaction with the Host

**DOI:** 10.3390/jof3040066

**Published:** 2017-12-01

**Authors:** Zhongming Li, Kirsten Nielsen

**Affiliations:** Department of Microbiology and Immunity, Medical School, University of Minnesota, 689 23rd Ave SE, Minneapolis, MN 55455, USA; zli89@jhmi.edu

**Keywords:** human fungal pathogen, morphology change, host-pathogen interaction, titan cell, spherules, hyphae

## Abstract

Morphological changes are a very common and effective strategy for pathogens to survive in the mammalian host. During interactions with their host, human pathogenic fungi undergo an array of morphological changes that are tightly associated with virulence. *Candida albicans* switches between yeast cells and hyphae during infection. Thermally dimorphic pathogens, such as *Histoplasma capsulatum* and *Blastomyces* species transform from hyphal growth to yeast cells in response to host stimuli. *Coccidioides* and *Pneumocystis* species produce spherules and cysts, respectively, which allow for the production of offspring in a protected environment. Finally, *Cryptococcus* species suppress hyphal growth and instead produce an array of yeast cells—from large polyploid titan cells to micro cells. While the morphology changes produced by human fungal pathogens are diverse, they all allow for the pathogens to evade, manipulate, and overcome host immune defenses to cause disease. In this review, we summarize the morphology changes in human fungal pathogens—focusing on morphological features, stimuli, and mechanisms of formation in the host.

## 1. Introduction 

Fungi are both ubiquitous and highly diversified. A “conservative” estimate for the number of fungal species is 1.5 million [1], with a recent estimate suggesting there may be over 5 million fungal species on earth [2]. About 100,000 species of fungi have been formally described [2]. Over 300 fungi have been shown to infect humans [3]. Most individuals in their lifetimes will contract superficial fungal infections. Fungal infections of the skin, hair, and nails are a common global problem. 20–25% of the world’s population have skin mycoses that are primarily caused by dermatophytes, or dermatophytosis [4]. Fungal infections of oral and genital tracts are also common. Mucocutaneous *C. albicans* infections are frequent in babies, immunocompromised individuals, diabetics, and obese individuals. In their childbearing years, 70–75% of women suffer from vulvovaginal candidiasis [5]. Invasive fungal infections have a much lower disease incidence than superficial infections, but invasive fungal infections cause unacceptably high mortality rates. About one and half million people die from invasive fungal infections annually [6], making them a significant global public health problem. While fungal diseases can affect healthy individuals, they pose a serious threat to immunocompromised individuals. Morphology switching is frequently associated with fungal pathogens and are tightly linked with virulence.

**Figure 1:**
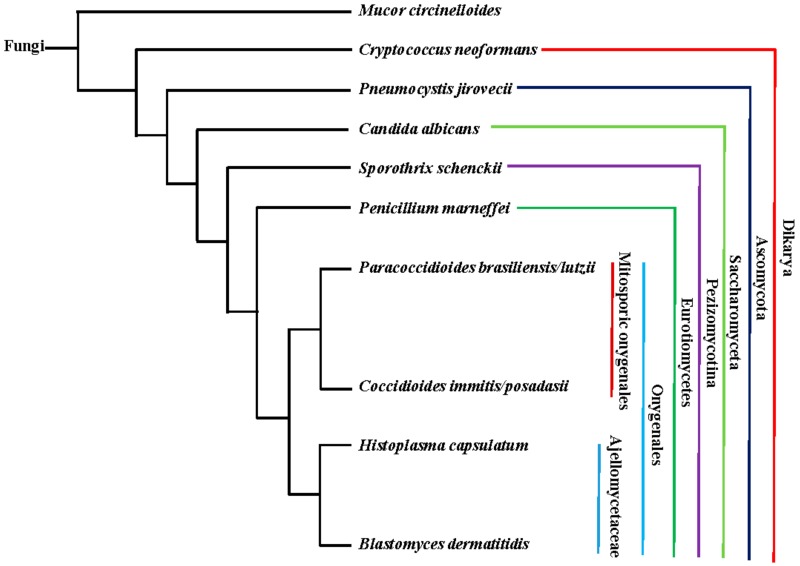
Evolutionary relationship between common human fungal pathogens that exhibit morphology changes. The tree was generated using Common Tree from the National Center for Biotechnology Information. *M. circinelloides* belongs to “Fungi *incertae sedis*”. *C. neoformans* is in the phylum Basidiomycota. The rest are in the phylum Ascomycota.

This review will discuss 12 common fungal pathogens that undergo morphology transitions upon interaction with the human host (Figure 1). The commensal fungus *Candida albicans* switches between yeast, pseudohyphae, and hyphae during infection and disease. Thermally dimorphic pathogens (*Histoplasma capsulatum*, *Blastomyces* spp., *Talaromyces marneffei*, *Coccidioides* spp., *Paracoccidioides* spp., *Sporothrix schenckii*, and others) transform from hyphal growth to yeast cells in response to elevated temperature. *Mucor circinelloides* changes from hyphae to multi-budded yeasts in anaerobic/high CO_2_ conditions. *Coccidioides* spp. and *Pneumocystis* spp. produce spherules and cysts, respectively, which allow for the production of offspring in a protected environment. Finally, *Cryptococcus* spp. suppress hyphal growth and instead produces an array of yeast cells—from large polyploid titan cells to small micro cells. These morphology changes dramatically influence the host-pathogen interaction and allow for these fungi to cause disease.

## 2. *C. albicans*—A Commensal Fungal Pathogen

**Figure 2:**
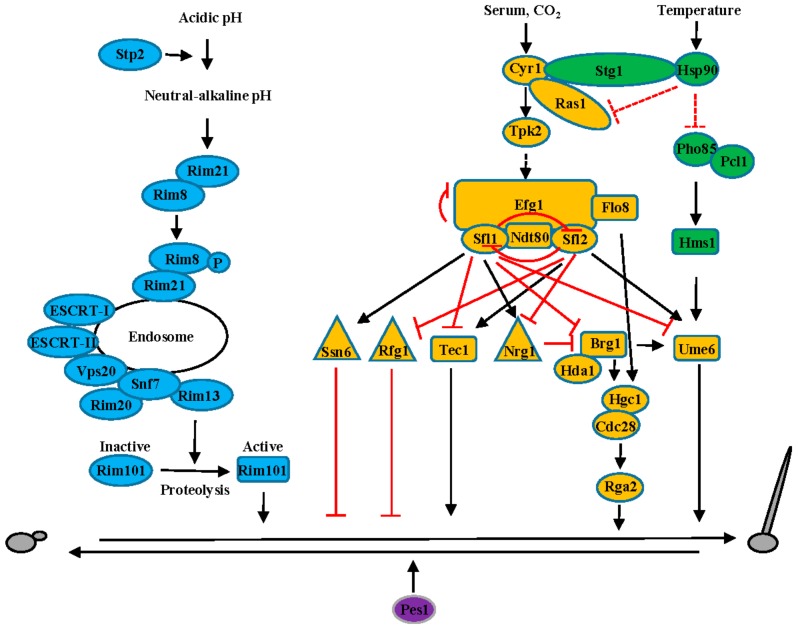
Regulation of the dimorphic transition in *C. albicans* via multiple signaling pathways. Signaling pathways are illustrated in different colors: cyclic AMP (cAMP)- protein kinase A (PKA) pathway (yellow), Hsp90 pathway (green), Rim101 pathway (blue) and Pes1 pathway (purple). Solid lines or arrows represent direct regulation. Dotted lines or arrows represent indirect or unknown regulation. Positive and negative relationships between components are illustrated in black and red, respectively. Transcription factors are illustrated with a rectangle, transcriptional co-repressors with a triangle, and other proteins with an oval.

*C. albicans* is a commensal fungal pathogen of humans that colonizes the skin and mucosal surfaces of most healthy individuals. *C. albicans* causes life-threatening infections in individuals with compromised immune systems. *C. albicans* exists as yeast, psuedophyphae, and hyphae, all of which are important for virulence. Yeast cells are essential for dissemination [7], while hyphal forms may be essential for invading mucosal surfaces. The morphology switch between budding yeast and hyphal growth is triggered by diverse host environmental cues, including temperature, pH, serum, and CO_2_ (Figure 2).

Signal transduction pathways that trigger morphogenetic changes in response to the host environment have been extensively studied in *C. albicans* (Figure 2)*.* The cyclic AMP (cAMP)—protein kinase A (PKA) pathway is the major pathway controlling hyphal growth induced by serum and CO_2_ in *C. albicans* [8,9]. Serum and CO_2_ act directly on the adenylate cyclase Cyr1 to activate the catalytic subunit of protein kinase A, Tpk2 [8,9,10]. Tpk2 then directly or indirectly phosphorylates the key transcription factor Efg1 [11,12,13]. Efg1 is a negatively auto-regulated transcription factor, which controls morphogenesis via interaction with heat shock factor-type transcriptional regulators Sfl1 and Sfl2, as well as transcription factors Ndt80 and Flo8 [11,12,13,14,15,16]. Sfl1 and Sfl2 antagonistically regulate morphogenesis in *C. albicans* [14]. Sfl1 acts via interaction with Efg1 and Ndt80 to repress expression of morphogenesis activators, such as transcription factors Ume6, Tec1, and Brg1, and Sfl2, while upregulating morphogenesis repressors, such as transcriptional co-repressors Ssn6 and Nrg1 [14,17,18,19,20,21,22]. In contrast, Sfl2 functions via Efg1 and Ndt80 to upregulate activators of hyphal growth, such as Ume6 and Tec1, while downregulating repressors of morphogenesis, such as the transcriptional co-repressor Nrg1 and Rfg1, as well as Sfl1 [14,18,19,21,22,23,24]. Nrg1 represses expression of its downstream target Brg1, which directly upregulates Ume6 and the hypha-specific G1 cyclin Hgc1 via interaction with the histone deacetylase Hda1 [24]. Hgc1 then activates the cyclin-dependent kinase Cdc28 to phosphorylate Rga2, a GTPase-activating protein of the central polarity regulator Cdc42 [25,26]. In addition, expression of Hgc1 is also controlled by Flo8, which functions via interaction with Efg1 [15,16].

In *C. albicans,* the molecular chaperone Hsp90 is a global regulator of morphogenesis in response to elevated temperature and acts by recruiting the co-chaperone Sgt1 to repress activity of Cyr1 [27]. Hsp90 also inhibits the activity of Cyr1 via direct or indirect interactions with Ras1 [28,29,30]. Finally, Hsp90 also controls morphogenesis by directly or indirectly repressing activity of Pho85^Pcl1^, which phosphorylates the transcription factor Hms1 to activate expression of Ume6 [18,31].

*C. albicans* senses alkaline pH via the receptor Rim21, which leads to the phosphorylation of the β-arrestin protein Rim8 [32]. This phosphorylated Rim8-Rim21 complex is taken up by endocytosis and recruits ESCRT-I, ESCRT-II, and Vps20-Snf7 [32,33]. Snf7 oligomerizes and recruits the scaffold protein Rim20 and the protease Rim13 to process the transcription factor Rim101 [34,35,36,37]. This processing activates Rim101 to promote transcriptional changes in genes that are required for the hyphal switch at alkaline pH [36,38]. *C. albicans* is also able to alters environmental pH, such as within the phagosome, to promote hyphal morphogenesis via the transcription factor Stp2. Stp2 upregulates expression of multiple amino acid permeases that are required for alkalinization of the phagosome [39].

While the transition from yeast to hyphae has been extensively studied in *C. albicans*, the switch from hyphae to yeast still remains poorly understood. Pes1, a *pescadillo* homolog in *C. albicans*, is involved in the hyphae to yeast switch, especially in the budding of yeast from lateral filamentous cells [40].

These detailed signal transduction pathway studies were recently expanded using genomic approaches. A genome-scale *C. albicans* mutant library was screened to identify additional regulators of morphogenesis in response to host-relevant environmental cues [41]. 872 mutants were identified with varying filamentation defects in response to serum [41]. Gene Ontology analysis found that many genes were involved in vesicle and intracellular protein transport, ergosterol biosynthesis, and N-linked glycosylation [41]. Many known morphogenetic regulators were identified, including Ras1, Hsp90, Cyr1, and Flo8 [41]. 102 genes encoding repressors of filamentation were identified, including 50 genes associated with cell cycle, such as the mitotic cyclin Clb2, the cyclin-dependent kinase Cdc28, members of the structural maintenance of chromosomes complex, as well as regulators of DNA replication [41].

Detailed analysis of signal transduction pathways in *C. albicans* have identified an intricate network that coordinates signals from diverse environmental cues to modify basic cellular functions, such as cell cycle, membrane and cell wall synthesis, and transport. Ultimately, these modifications allow for *C. albicans* to seamlessly transition between yeast and hyphal growth throughout its interaction with the host.

## 3. Thermally Dimorphic Fungal Pathogens

**Figure 3:**
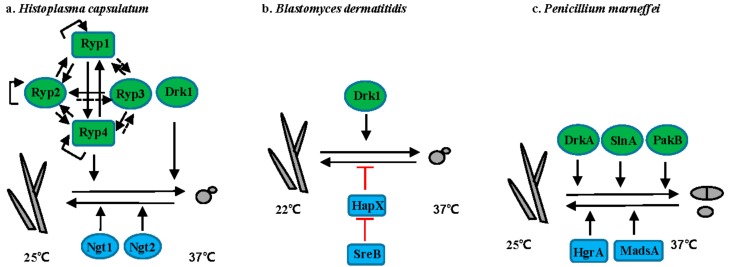

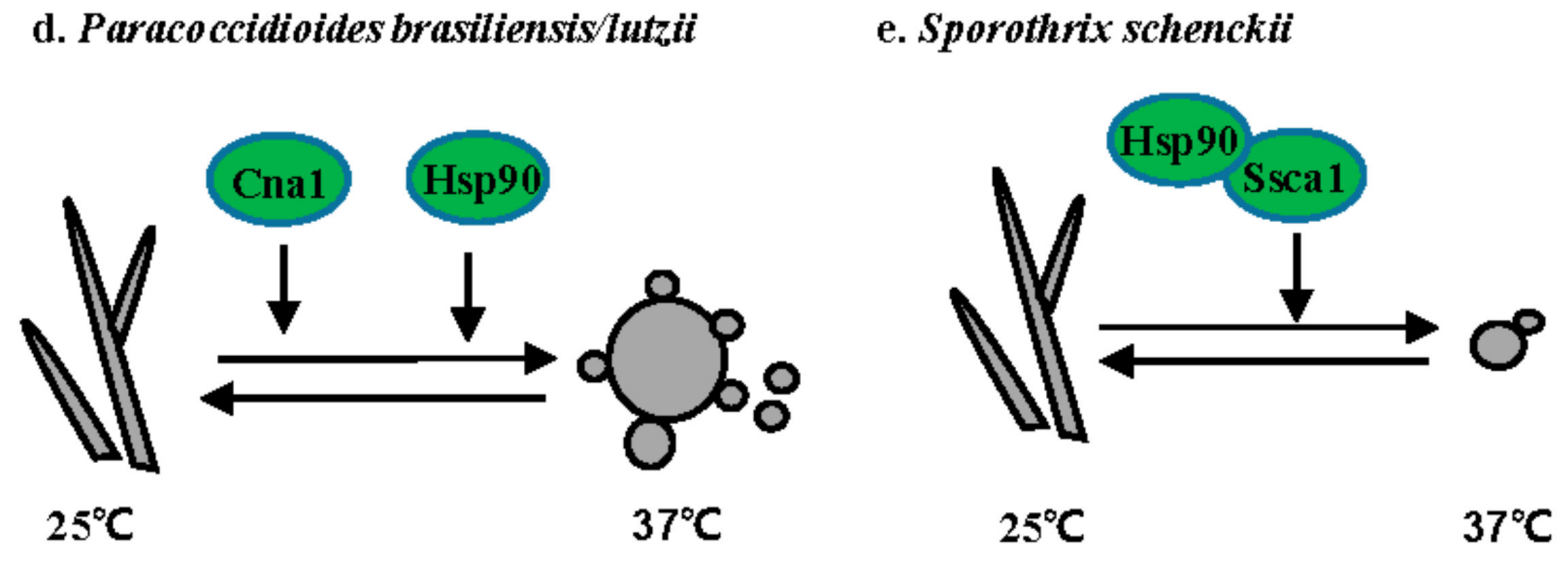
Regulation of the morphogenetic transition in thermally dimorphic fungal pathogens. (**a**) *H. capsulatum*; (**b**) *B. dermatitidis*; (**c**) *T. marneffei*; (**d**) *P. brasiliensis*; and, (**e**) *S. schenckii*. Solid lines or arrows represent direct regulation. Dotted lines or arrows represent indirect or unknown regulation. Positive and negative relationships between components are illustrated in black and red, respectively. Transcription factors are illustrated as rectangles, and other proteins as ovals. Proteins controlling hyphal to yeast transitions are indicated in green. Proteins controlling yeast to hyphal transitions are indicated in blue.

**Figure 4:**
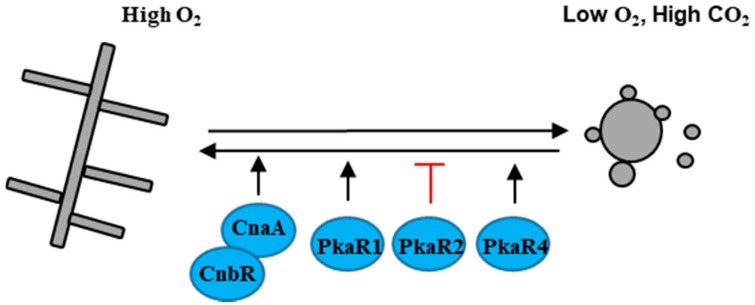
Dimorphic transition of *M. circinelloides* in response to O_2_/CO_2_. Positive and negative relationships between components are illustrated in black and red, respectively.

Thermally dimorphic human pathogenic fungi represent a group of fungi that grow in hyphal form in the environment but shift to yeast form within the host (Figure 3 and Figure 4). For many of these organisms, elevated temperature is one of the main host signals that initiate the hyphal to yeast conversion (Figure 3).

### 3.1. Histoplasma Species

*Histoplasma* spp. cause life-threatening histoplasmosis, predominantly in immunocompromised individuals. Histoplasmosis occurs worldwide, with *H. duboissii* endemic in Africa, whereas *H. capsulatum* is mainly endemic in the Ohio and Mississippi river valleys in the United States, South America, Southeast Asia, and Africa [42]. *H. capsulatum* grows as filamentous cells at 25 °C, while at 37 °C it converts into budding yeast (Figure 3a). Transition from hyphae to yeast is positively controlled by the hybrid histidine kinase Drk1 [43]. In addition, three transcriptional regulators—Ryp1, Ryp2, Ryp3, and Ryp4—are required for the switch from hyphae to yeast at 37 °C [44,45,46]. All four Ryp proteins require each of the others for their expression and directly interact with *RYP1*, *RYP2*, and *RYP4* promoters in response to elevated temperature [46]. Although none of the Ryp proteins directly associate with the *RYP3* promoter, *RYP3* expression level still depends on other Ryp proteins. Acting opposite to temperature, N-acetylglucosamine (GlcNAc) promotes the transition from yeast to hyphae via the GlcNAc transporters Ngt1 and Ngt2 in *H. capsulatum* at 25 °C [47].

### 3.2. Blastomyces Species

Blastomycosis is caused by *B. dermatitidis*, *B. gilcristii*, and *B. percursus.* These species are endemic in the Ohio-Mississippi and St. Lawerence river valleys, southeastern US, Great Lakes region, and Canada [48]. *B. dermatitidis* grows as a mold at 22 °C and as yeast at 37 °C (Figure 3b). Like in *H. capsulatum*, the hybrid histidine kinase Drk1 is required for transition from mold to yeast and strains with reduced *DRK1* expression are avirulent [43]. Transition from yeast to mold in *B. dermatitidis* is controlled by two transcription factors SreB and HapX [49,50]. Mutation of *SREB* or overexpression of *HAPX* inhibit the yeast-to-mold transition [50]. SreB negatively controls the expression of *HAPX* via direct interaction with the promoter of *HAPX*, although the defect in SreB null mutants is thought to be related to impaired biosynthesis of neutral lipids and not directly to *HAPX* expression [50].

### 3.3. T. marneffei

*T. marneffei*, an emerging human-pathogenic fungus endemic to Southeast Asia, causes severe, and often deadly, infection in immunocompromised patients. At 25 °C, *T. marneffei* grows as filamentous hyphae and undergoes asexual development to produce infectious conidia. At 37 °C, *T. marneffei* grows as yeast cells that replicate by fission (Figure 3c). The two-component histidine kinases DrkA and SlnA are involved in morphology switching, as overexpression of DrkA or SlnA promotes transition from hyphal to yeast growth even at 25 °C, whereas the deletion of DrkA or SlnA results in yeast growth at 37 °C in vitro [51]. Interestingly, PakB, encoding a p21-activated kinase is required for yeast morphogenesis within macrophages, but not during yeast growth in vitro at 37 °C, suggesting PakB is involved in the signaling pathway that controls the dimorphic switch in response to host stimuli, not temperature [52]. Additionally, two transcription factors MadsA and HgrA are required for the dimorphic switch from yeast to hyphae [53,54]. Overexpression of *MADSA* can induce hyphal growth at 37 °C [53]. Similarly, deletion of *HGRA* causes a defect in the dimorphic transition from yeast to hyphae [54].

### 3.4. Paracoccidioides Species

*P. brasiliensis* and *P. lutzii* are temperature-dependent dimorphic fungi that cause paracoccidioidomycosis (PCM), the most prevalent human deep mycosis in Latin America [55]. *P. brasiliensis* grows as hyphae at 25 °C and as yeast with multiple buds at 37 °C or in the host (Figure 3d). Both the calcineurin catalytic subunit Cna1 and Hsp90 are involved in the hyphal to yeast transition. Treatment with either the calcineurin inhibitor cyclosporine A or the Hsp90 inhibitor geldanamycin prevents the hyphal to yeast transition [56,57].

### 3.5. S. schenckii

*S. schenckii* is a dimorphic fungus that causes sporothrichosis worldwide [58]. Sporotrichosis is usually acquired via traumatic inoculation with contaminated plant debris, thorns, and soil [58]. *S. schenckii* grows as hyphae at 25 °C and as yeast at 37 °C (Figure 3e). The calcium/calmodulin kinase 1 Sscm1 is involved in the hyphal to yeast transition via direct interaction with Hsp90 [59]. A *DRK1* homolog has also been identified in *S. schenckii*. While the role of Drk1 has not been directly dissected in *S. schenckii*, it is upregulated 24-fold in yeast when compared to hyphae.

### 3.6. Summary 

Recent studies show that multiple signaling pathways control morphogenetic switches in thermally dimorphic fungal pathogens, including two-component signaling pathways, Hsp90 and calcium signaling. In addition, an array of novel transcription factors are critical for hyphal to yeast transitions. For many of the thermally dimorphic fungal pathogens, the upstream and downstream components of these signaling pathways and their link to the transcription factors remains unknown. Finally, temperature is not the only stimulus for morphology change during these host-pathogen interactions. Future research on the mechanisms by which the thermally dimorphic fungal pathogens sense and respond to the host environment will provide invaluable insight into aspects of the dimorphic switch specific to interactions with the host.

## 4. Aerobically/Anaerobically Dimorphic Fungal Pathogen

*M. circinelloides* is the causal agent of the fungal infection mucormycosis, an uncommon but frequently lethal fungal infection of humans. *M. circinelloides* usually grows as hyphae aerobically, and as multi-budded yeast in anaerobic conditions [60] (Figure 4). *M. circinelloides* encodes three protein kinase A regulatory subunits PkaR1, PkaR2, and PkaR4 that play different roles in dimorphic transition [61,62,63]. Over-expression of *PKAR1* promotes filamentation and branching, whereas the mutation of *PKAR1* leads to a defect in the yeast to hyphal transition [61,62]. PkaR4 is essential in *M. circinelloides*, but the hererokaryon mutant *pkaR4* shows a defect in germ tube emergence upon shift from anaerobic to aerobic growth, indicating that PkaR4 also promotes the yeast to hyphal transition [63]. Unlike PkaR1 and PkaR4, mutation of *PKAR2* promotes the transition from yeast to hyphae [63]. Additionally, inhibition of the calcineurin catalytic A subunit CnaA or mutation of the calcineurin regulatory B subunit *CNAR* results in cells locked in the yeast phase, indicating that calcinurin governs the dimorphic transition in *M. circinelloides* [64].

*M. circinelloides* is an emerging zygomycete fungal pathogen, and the current status of research on dimorphic switches in *M. circinelloides* is in its infancy. However, the genetic and molecular tools have been well developed in *M. circinelloides*, which will promote the advancement in understanding the dimorphic transition and its roles in the pathogenesis of *M. circinelloides.*

## 5. Fungal Pathogens That Exhibit Cell Size Variation

**Figure 5:**
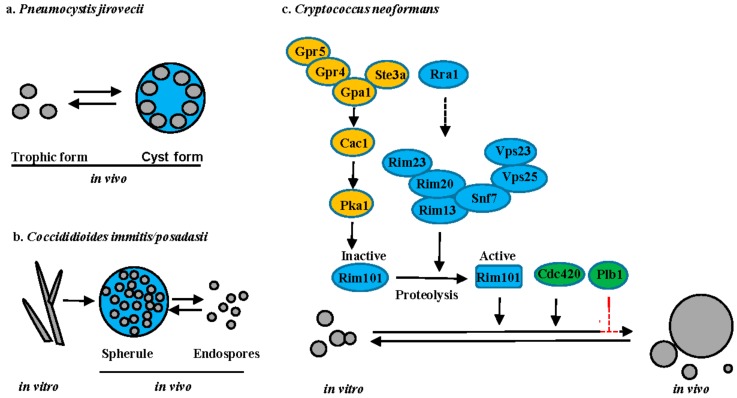
Changes in cell size upon interaction with host. (**a**) *P. jirovecii*; (**b**) *C. immitis*; and, (**c**) *C. neoformans*. Signaling pathways are illustrated with different colors: cAMP-PKA (yellow), Rim101 (blue) and others (green). Solid lines or arrows represent direct regulation. Dotted lines or arrows represent indirect or unknown regulation. Positive and negative relationships between components are illustrated in black and red, respectively. Transcription factors are illustrated as rectangles and other proteins illustrated as ovals.

*Pneumocystis* spp., *Coccidioides* spp., and *Cryptococcus* spp. all exhibit changes in cell size during infection (Figure 5). However, each has its own unique morphological characteristics. *Pneumocystis* spp. form small trophic cells and larger cysts. *Coccidioides* spp. are not only thermally and aerobically dimorphic, but also generate large spherules. Finally, while *Cryptococcus* spp. can grow both as hyphae (during sexual development) and yeast in the environment, only yeast are observed in vivo. These *Cryptococcus* yeast can differentiate into both small micro cells and large polyploid titan cells.

### 5.1. Pneumocystis Species 

*Pneumocystis* spp. are host organism specific with *P. carinii* infecting rats, *P. murina* infecting mice and *P. jirovecii* infecting humans. *P. jirovecii* causes a severe pneumonia (Pneumocystis pneumonia or PCP) in immunocompromised individuals worldwide. Microscopic analysis of tissue sections has revealed that *Pneumocystis* has two predominant life-cycle forms: the trophic form and the cyst form (Figure 5a). The trophic form can replicate either sexually or asexually. During sexual reproduction, two haploid trophic forms conjugate to produce an early cyst with two nuclei. The nuclei undergo meiosis to form intermediate cyst with four nuclei. The nuclei in the intermediate cyst undergo mitosis to produce the mature cyst containing eight haploid trophic cells. The cyst ruptures to release the trophic cells that either reproduce asexually via mitosis or re-enter the sexual cycle. [65]. A functional beta-1,3-d-glucanase is exclusively expressed in cysts [66]. Interestingly, cysts are thought to be the infected form of *Pneumocystis*, based on the rodent model of PCP [67]. Unfortunately, the molecular mechanisms underlining the troph to cyst morphology change and the role of these two morphologies during the host-pathogen interaction remain largely unknown. A reproducible genetic system in either *P. carinii* or *P. murina* is needed to allow for the identification of the molecular signals underlying these morphology changes and their unique role in both virulence and transmission.

### 5.2. Coccidioides Species 

*C. immitis* and *C. posadasii* are dimorphic pathogenic fungi that cause coccidioidomycosis, also known as Valley fever in the southwestern United States, parts of Mexico, and South America [68]. *Coccidioides* spp. are thermally dimorphic and evolutionarily closely related to other thermal dimorphs [69]. However, *Coccidioides* can produce large spherules upon inhalation, in which yeast-like endospores develop (Figure 5b). Mature spherules are too large (30–80 µm) to be phagocytosed, resulting in the hypothesis that they provide a protected environment for cellular replication [69]. Upon rupture, endospores that are produced within the spherule are released and can survive within phagocytes [69]. Dissemination of *Coccidioides* from the lungs to secondary tissues is thought to be via the endospores. Interestingly, aerobic/anaerobic conditions (CO_2_ tension) and estradiol are important for spherule maturation, but the underlying molecular pathways involved in spherule production are still largely unknown.

### 5.3. Cryptococcus Species 

Disease that is due to *C. neoformans* is predominantly observed in immunocompromised individuals, whereas *C. gattii* can cause disease in both immune-compromised and apparently healthy individuals. In vitro, *Cryptococcus* cells typically grow as round yeast cells, ranging in size from 4–7 µm. However, *C. neoformans* exhibits a vast diversity of cell sizes in vivo, particularly in the lungs, with cell sizes ranging from 1 to over 100 µm in cell body diameter [70,71,72,73,74] (Figure 5c). Recent studies have shown that cells with different sizes have distinct traits, resulting in classification of some of the cell types, such as micro cells and titan cells [70,71,72,73,74,75]. The most well studied are the titan cells that are generally larger than 10 µm in cell body diameter. Beyond their large size, titan cells have several unique characteristics when compared to typical cells that are grown in vitro, such as polyploidy, thicker cell wall with high chitin content, and a dense highly cross-linked capsule [73,74,76]. These novel traits promote survival and dissemination of *C. neoformans* during infection by limiting phagocytosis by host macrophages, increasing resistance to oxidative, and nitrosative stresses, triggering a detrimental Th2 immune response, and producing daughter cell progeny with genomic diversity [74,76,77,78]. Titan cell formation is regulated by the pheromone signaling pathway, the cAMP/PKA pathway, and the Rim101 pathway [73,74,79,80,81,82]. Both the pheromone receptor Ste3a and the G-protein coupled receptor Gpr5 can independently stimulate titan cell production via interaction with the G-protein Gpa1 to activate the adenylate cyclase Cac1, which then activates Pka1 to phosphorylate the transcription factor Rim101 [73,74,80,81,82]. Activation of Rim101 requires both phosphorylation by Pka1 and proteolysis by the proteolysis complex [81,82]. As in *C. albicans*, activation of the proteolysis complex requires assembly of ESCRT-I Vps23, ESCRT-II Vps25, and ESCRT-III Snf7 as a scaffolding platform [82]. Recently, a novel transmembrane protein Rra1 was reported to act upstream of Rim23, likely functioning as a sensor similar to the Rim21 sensor in *C. albicans.* The predictive structure of Rra1 contains 7-transmembrane domains, similar to Rim21 [82]. Additionally, both Cdc420 and phospholipase B (Plb1) have been implicated in titan cell formation, but their mode of action remains unclear [80,83].

### 5.4. Summary

In addition to traditional hyphal to yeast dimorphic transitions, some of the human pathogenic fungi also exhibit unique morphology changes that impact pathogenesis. Some of these morphology changes are associated with cell size and ploidy changes. The large size of *Cryptococcus* titan cells and *Coccidioides* spherules both protect from phagocytosis, and thus may serve as protective structures to reduce impact of innate immune responses. *Cryptococcus* titan cells, *Pneumocystis* cysts, and *Coccidioides* spherules are also all polypoid. Polyploid cells are very common in nature and are found in plants, animals, and arthropods [84]. Polyploid cells are usually formed through endoreplicative cycles [84], thus it is likely that endoreplicative cycles generate titan cells and spherules. *Pneumocystis* has differential activity of a Cdc2/Cdc13 cyclin-dependent kinase complex over the life cycle, with greater activity in cysts when compared with the trophic forms, also supporting a link between cell cycle and ploidy variation [85]. Yet the exact nature of the cycle cycle alterations in *Cryptococcus*, *Pneumocystis*, and *Coccidioides* that generate the large polyploidy structures remain unknown. Further, how differences in these cell cycles lead to the different structures produced by these human pathogenic fungi and their subsequent daughter cells also remain to be explored.

## 6. Conclusions

As described above, the human fungal pathogens undergo diverse morphology changes in response to the host environment. These morphology changes enable the human fungal pathogens to rapidly adapt to and survive not only the high temperature and low oxygen environment of the host, but also protect against the host immune response. With recent developments in genetic, genomic, and post-genomic tools, the signaling pathways that are involved in these morphological transitions have begun to be elucidated using gene knockout or knockdown, transcriptomic, and proteomic studies. While the human fungal pathogens exhibit diverse morphologies, many commonalities in the underlying mechanisms that are controlling these morphological transitions have been uncovered. For example, elevated temperature induces morphology transitions in *C. albicans* and the thermally dimorphic human fungal pathogens. Similarly, CO_2_ triggers morphological switchs in *C. albicans*, *M. circinelloides*, and *Coccidioides immitis*. The cAMP/PKA and Hsp90 pathways are used to control morphological responses in many of the human pathogenic fungi.

Given the importance of morphological transitions in the pathogenesis of the human fungal pathogens, more extensive and more detailed studies are needed to gain deeper insights into the commonalities between the morphologies and their function during infection. Ultimately, future studies addressing how human fungal pathogens control morphology and virulence in response to host environmental cues will provide information that could lead to the development of novel treatment strategies, which are aimed at blocking these important morphological changes.
